# *Borrelia burgdorferi* sensu lato in humans in a rural area of Paraná State, Brazil

**DOI:** 10.1590/S1517-838246220140097

**Published:** 2015-06-01

**Authors:** Daniela Dib Gonçalves, Rodrigo Assunção Moura, Mônica Nunes, Teresa Carreira, Odilon Vidotto, Julio Cesar Freitas, Maria Luísa Vieira

**Affiliations:** 1 Universidade Estadual de Londrina, Programa de Pós-gradução em Ciência Animal, Universidade Estadual de Londrina, Londrina, PR, Brasil, Programa de Pós-gradução em Ciência Animal, Universidade Estadual de Londrina, Londrina, PR, Brazil.; 2 Universidade Paranaense, Medicina Veterinária Preventiva, Universidade Paranaense, Umuarama, PR, Brasil, Medicina Veterinária Preventiva, Universidade Paranaense, Umuarama, PR, Brazil.; 3 Universidade Nova de Lisboa, Medical Microbiology Unit, Institute of Hygiene and Tropical Medicine, Universidade Nova de Lisboa, Lisbon, Portugal, Leptospirosis and Lyme Borreliosis Group, Medical Microbiology Unit, Institute of Hygiene and Tropical Medicine, Universidade Nova de Lisboa, Lisbon, Portugal.; 4 Universidade Estadual de Londrina, Departamento de Medicina Veterinária Preventiva, Universidade Estadual de Londrina, Londrina, PR, Brasil, Departamento de Medicina Veterinária Preventiva, Universidade Estadual de Londrina, Londrina, PR, Brazil.

**Keywords:** Brazil, human, lyme borreliosis, PCR, sequencing

## Abstract

This study describes the detection of Borrelia garinii and *Borrelia burgdorferi* sensu stricto (s.s.) in Brazilian individuals using PCR and DNA sequencing. Our results suggest that these species are emerging pathogens in this country, and additional studies are necessary to determine the epidemiological characteristics of this disease in Brazil.

Lyme borreliosis (LB) is a tick-borne disease caused by genospecies of the *Borrelia burgdorferi* sensu lato (s.l.) complex (Steere, 1997). The genospecies causing LB vary according to the geographic region: *B. andersonii,* is mainly found in North America, *B. afzelli* and *B. garinii* in Europe, *B. japonica* in Japan, and *B. burgdorferi* sensu stricto (s.s.) has been detected on several continents (Qiu *et al.*, 2008; Rudenko *et al.*, 2011). Migratory birds cause the dissemination of *Borrelia* spp. between continents, and the establishment and maintenance of these spirochetes in a new environment depends on the presence of their reservoir hosts (tick species) and host-vector interactions (Hasle, 2013; Norte *et al.*, 2013).

In Europe and North America, *B. burgdorferi* genospecies causing LB are mainly transmitted by the tick Ixodes ricinus (Steere, 1997; Qiu *et al.*, 2008; Rudenko *et al.*, 2011). In contrast, in Brazil, some studies have indicated the presence of these spirochetes in ticks from the Amblyomma, Rhipicephalus and Dermacentor genera, demonstrating the need for further studies to determine the vectors able to transmit LB in this country (Yoshinari *et al.*, 2010; Gonçalves *et al.*, 2013; Montovani *et al.*, 2013).

In Brazil, this disease, which is known as Brazilian lyme-like disease or Baggio-Yoshinari syndrome, has been poorly studied (Yoshinari *et al.*, 2010). Therefore, its epidemiology and most prevalent genospecies are not well defined (Dantas-Torres, 2008; Steps *et al.*, 2009). Cases of this disease have been detected in humans and animals by serologic methods and/or by clinical symptoms in the northern (Amazonas and Tocantins States) (Abel *et al.*, 2000; Carranza-Tamayo *et al.*, 2012), midwestern (Mato Grosso do Sul State) (Costa *et al.*, 2002; Naka *et al.*, 2008), southeastern (Espírito Santo, Rio de Janeiro and São Paulo States) (Azulay *et al.*, 1991; Passos *et al.*, 2009; Yoshinari *et al.*, 2003, 2010) and southern (Paraná State) (Gonçalves *et al.*, 2013a, 2013b) regions of Brazil. Most of the cases affecting humans have been detected in inhabitants of rural areas, where the incidence of this zoonosis is high due to the close proximity of humans to the animal population, which are often parasitized by ticks.

Despite these findings, studies have reported negative serology in most of the individuals showing clinical signs of this disease and have failed to define its etiologic agent (Yoshinari *et al.*, 2010). Studies involving this pathogen in Brazil have mainly assessed serology; thus, the aim of this study was to use molecular methods to determine the particular species of the *B. burgdorferi* s.l. complex that are present in humans in a small rural area in the northern region of Parana State, Brazil.

From February to November 2007, blood samples were collected voluntarily from 207 asymptomatic humans between 15 and 72 years of age living on 63 small rural properties in the northern region of Parana State. These residents also worked on family farms with animals.

After collection, the blood samples were forwarded to the Leptospirosis Laboratory of Preventive Veterinary Medicine Department at Universidade Estadual de Londrina (UEL) to obtain serum samples. Each sample was kept in a sterile container and stored at −20 °C until its use in the molecular tests, which were performed at the Laboratory of Leptospirosis and Lyme Borreliosis, Medical Microbiology Unit, Institute of Hygiene and Tropical Medicine (IHMT), Universidade Nova de Lisboa (UNL), Portugal.

DNA from the serum samples and the *B. garinii* culture (strain PBi), which contained approximately 2 × 10^7^ cells/mL (used as a positive control), was extracted using the Puregene™ Gentra Cell & Tissue Kit (Qiagen, Valencia, CA, USA) according to the manufacturers protocol. The extracted DNA was stored at −20 °C until further use.

The detection of the *B. burgdorferi* s.l. complex genospecies was performed by nested PCR targeting the 5S(*rrf*)-23S(*rrl)* intergenic spacer region, as previously described (Schwartz *et al.*, 1992; Postic *et al.*, 1994; Chao *et al.*, 2011). Amplicons of ~226–266 bp, depending on the strain of *Borrelia* spp., were purified, and both strands were directly sequenced by the Macrogen Sequencing Service, Inc. (Seoul, Korea). The primers used for DNA amplification were also used for sequencing. The results obtained were compared with existing Borrelia genospecies sequences in the GenBank database using the BLAST sequence analysis tool (http://blast.ncbi.nlm.nih.gov/Blast.cgi).

An epidemiological survey was also conducted using a structured questionnaire, in which the participants answered questions regarding risk factors related to the disease, including education level, the presence of domestic animals (dogs, cats and others), wild animals and rodents at the rural property, the presence of ticks attached to the body and the observation of ticks inside homes (Gonçalves *et al.*, 2013).

The data obtained from the epidemiological survey were statistically analyzed using Yates or Fisher's exact test with a chi-square (χ^2^) correction. The tabulations of the epidemiological data and analyses were performed using the EpiInfo statistical program version 6.04 (CDC) at a 5% significance level. As an association measure, odds ratios (ORs) were calculated with confidence intervals of 95% (Dean *et al.*, 1994).

Of the 207 human serum samples analyzed, two (0.96%) showed positive nested-PCR results for the *B. burgdorferi* s.l. complex with amplicon sizes of ~230 bp. The BLAST analysis showed high sequence similarity (100%) with two different Borrelia genospecies. The nucleotide sequences obtained have been submitted to the GenBank database under the accession numbers KF790698 and KF790699. One strain (J-70) was identified as *B. garinii*, and the other strain (J-96) was identified as *Borrelia burgdorferi* s.s. (Figure 1A and 1B). Both samples were identified in males (15 and 72 years old, respectively) who worked with different animal species and performed various functions, such as assisting with births and slaughtering and castrating cattle. The analysis of the variables associated with the presence of *Borrelia burgdorferi* s.l. DNA is shown in Table 1.

**Figure 1:**
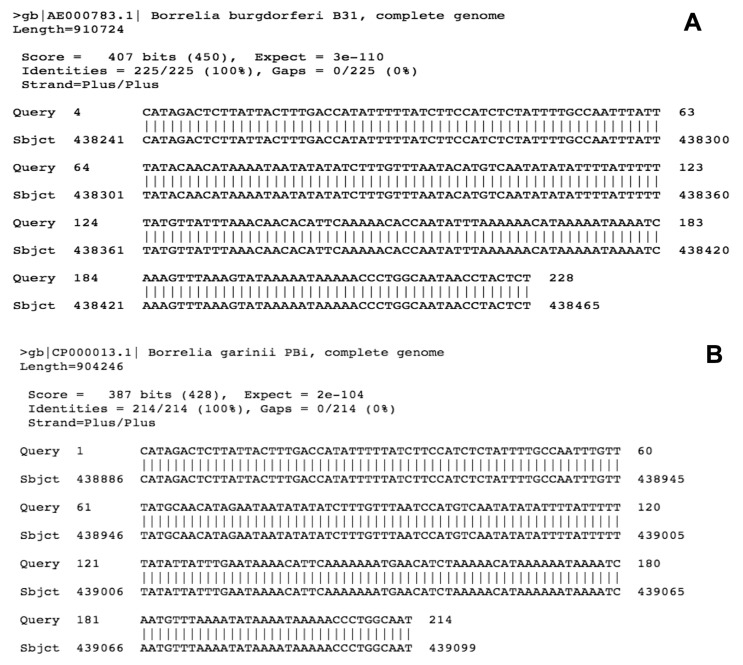
BLAST sequence analysis. (A) Alignment comparison of sequences generated from serum sample J-96 with the 5s(rrf)-23s (rrl) intergenic spacer region of *Borrelia burgdorferi* B31 strain (AE000783.1); (B) Alignment comparison of sequences generated from serum sample J-70 with the 5S (*rrf*)-23S (*rrl*) intergenic spacer region of *Borrelia garinii* (Pbi strain) (CP000013.1).

**Table 1:** Variables associated with the presence of DNA *Borrelia burgdorferi* s.l. in serum samples from 207 residents of the rural area of Jataizinho (PR), 2007.

Disease variables	Positive DNA total (%)	p value	OR CI ( 95%)
Lyme Borreliosis			
Ticks attached to the body			
Yes	02/16 (12.50)	0.0056*	
No	00/191 (0.00)		
Presence of ticks inside of house			
Yes	02/27 (7.40)	0.0164*	
No	00/180 (0.00)		

p = probability;

* Fisher's exact test;

OR = Odds ratio; CI = Confidence interval (Gonçalves *et al.*, 2013).

The two nested PCR-positive serum samples for *B. burgdorferi* s.l. in this study have also been detected by indirect immunofluorescence assay (IFA) and western blot (WB) in a previously published study (Gonçalves *et al.*, 2013a).

Brazilian lyme-Like disease, or Baggio-Yoshinari syndrome, was first reported in Brazil in 1992, but the causative agent of *Borrelia* infection has not been isolated or identified to date (Yoshinari *et al.*, 2003, 2010). Many aspects of the disease, such as the symptoms and frequency of recurrence after treatment, appear to differ in Brazilian individuals compared with those inhabiting the northern hemisphere (Yoshinari *et al.*, 2010). Moreover, *Amblyomma cajennense* and *Rhipicephalus microplus* ticks are believed to be involved in the transmission cycle of *B. burgdorferi* s.l. (Barros-Battesti *et al.*, 2000; Yoshinari *et al.*, 2003; Yparraguirre *et al.*, 2007).

Researchers from different countries have detected *B. burgdorferi* s.l. DNA in ticks of the *Dermacentor* (Gonçalves *et al.*, 2013b; Lledó *et al.*, 2014), *Ixodes* (Leyedet *et al.*, 2014; Morshed *et al.*, 2006; Hjgaard *et al.*, 2014; Dingler *et al.*, 2014; Prusinki *et al.*, 2014; Masuzawa *et al.*, 2014; Barbieri *et al.*, 2013) and *Rhipicephalus* (Maia *et al.*, 2014; Niu *et al.*, 2014) genera, which parasitize humans and different animal species. These studies have contributed to the understanding of borreliosis epidemiology, as they have indicated the main vectors involved in the transmission of this disease according to the region studied.

The presence of *Borrelia burgdorferi* s.l. was detected in Brazilian individuals by serological and molecular tests. Different researchers have demonstrated the presence of antibodies against *B. burgdorferi* s.s. and *B. garinii* by WB and/or ELISA tests in symptomatic and asymptomatic humans with histories of contact with ticks in Brazil (Costa *et al.*, 2002; Naka *et al.*, 2008; Gonçalves *et al.*, 2013a).

A recent study in Brazil detected the *flgE* gene from *B. burgdorferi* by PCR and DNA sequencing in three peripheral blood samples collected from humans with clinical symptoms of borreliosis and histories of tick exposure (Mantovani *et al.*, 2012). Gonçalves *et al.* (2013b) also detected the presence of these bacteria in Brazil using PCR and DNA sequencing, indicating that the detected DNA sequences in two ticks of the *Dermacentor nitens* species shared 99.99% homology with the *B. burgdorferi* sensu stricto (s.s.) strain B31. Despite these findings, further studies are necessary to delineate the presence of this pathogen in Brazil.

In the present study, *B. garinii* and *B. burgdorferi* s.s. were detected by molecular methods for the first time in residents of rural areas, who were directly or indirectly exposed to wild and/or domestic animals and ticks in the northern region of Parana State, confirming the presence of these genospecies in Brazil. The variables studied, such as the presence of ticks inside homes (p = 0.0164) and the presence of ticks attached to the body (p = 0.0056), were significant when associated with the *B. burgdorferi* s.l. DNA findings. These data are in accordance with other studies, which have also associated tick exposure with illness in humans by serological techniques (Yoshinari *et al.*, 2003, 2007; Mantovani *et al.*, 2012; Gonçalves *et al.*, 2013a).

However, the low frequency of *Borrelia* genospecies observed can be justified if these species are emerging pathogens in the country due to the dissemination of *B. burgdorferi* s.l. by migratory birds, and this hypothesis should not be discarded (Yoshinari *et al.*, 2010; Hasle, 2013).

Studies of the Brazilian lyme-Like disease, or Baggio-Yoshinari syndrome, have revealed differences in epidemiological, clinical and laboratorial characteristics compared with those reported in affected individuals in the northern hemisphere, suggesting the existence of differing etiological agents in the two locations (Yoshinari *et al.*, 2010). In Brazil, despite the wide geographical distribution of both invertebrate and vertebrate hosts for *Borrelia* spp., there are few descriptions of these spirochetes. Thus, further serological and molecular studies are needed in humans, different species of domestic and wild animals, and ticks, in particular, to better understand the epidemiology of *Borrelia* spp.

## Ethics Committee

This research was approved by the Committee of Ethics in Research involving Humans (CEP) from the State University of Londrina (UEL) (No. 319/06).
